# Study and Application on the Electromagnetic Stainless Steel: Microstructure, Tensile Mechanical Behavior, and Magnetic Properties

**DOI:** 10.3390/ma17122998

**Published:** 2024-06-19

**Authors:** Che-Wei Lu, Fei-Yi Hung, Tsung-Wei Chang, Ho-Yen Hsieh

**Affiliations:** 1 Department of Materials Science and Engineering, National Cheng Kung University, Tainan 70101, Taiwan; n56121115@gs.ncku.edu.tw; 2 Electric Motor Technology Research Center, National Cheng Kung University, Tainan 70101, Taiwan; n2897109@gmail.com; 3 Walsin-Lihwa Corporation, Tainan 73743, Taiwan; heyen_hsieh@walsin.com

**Keywords:** 430 stainless steel, microstructure, mechanical properties, magnetic anneal, hysteresis curve

## Abstract

Stainless steel grade 430 is a type of soft magnetic electromagnetic material with rapid magnetization and demagnetization properties. Considering the delay phenomenon during operation, this study selected 430 stainless steel as the material and explored various metallurgical methods such as magnetic annealing and the addition of Mo as well as increasing the Si content to investigate the microstructure, mechanical behavior, and magnetic properties of each material, aiming to improve the magnetic properties of 430 stainless steel. Experimental results showed that the four electromagnetic steel materials (430F, 430F-MA, 434, and KM31) had equiaxed grain matrix structures, and excellent tensile and elongation properties were observed for each specimen. Additionally, the magnetic properties of the 430F specimen were similar under the DC and AC-10 Hz conditions. According to the hysteresis curves under different AC frequencies (10, 100, 1000 Hz), both magnetic annealing and the addition of Mo could reduce the Bm, Br, and Hc values of the raw 430F material. Increasing the Si content resulted in a decrease in Hc values and an increase in Bm and Br values.

## 1. Introduction

Stainless steel is a type of steel alloy wherein the chromium content in the base composition exceeds 10.5 wt.%, while the carbon content is below 1.2 wt.%. Various trace elements such as nickel, molybdenum, silicon, etc. are added to achieve anti-oxidative properties, corrosion resistance, and suitable mechanical properties [1,2]. With numerous types available, stainless steel is a multifunctional metal material widely employed in different sectors including the food, medical, and industrial fields, each serving distinct purposes [3,4].

Electrical steel is a type of soft magnetic material characterized by rapid magnetization and demagnetization, primarily utilized in magnetic circuits within electrical machinery. It lacks inherent magnetism but exhibits magnetic properties upon electrification, commonly employed in the manufacturing of transformers, motors, generators, and other electrical devices for converting electrical energy into secondary electrical energy or mechanical energy [3,4]. Soft magnetic stainless steels approach the magnetic properties of industrial pure iron and possess the excellent dynamic magnetic characteristics and corrosion resistance. It is easily magnetized and demagnetized, featuring low coercive force and high magnetic permeability, thus maintaining stable magnetic performance under high temperature and high-frequency conditions. The magnetic properties of soft magnetic stainless steel vary under different electrical current conditions, leading to diverse requirements and application scopes. Presently, there is a lack of systematic exploration in the literature regarding the relationship between electrical current conditions (DC or AC or frequency) and the magnetic properties of soft magnetic stainless steel [5].

The 430 stainless steel belongs to the category of soft magnetic stainless steel, primarily composed of iron, carbon, chromium, and other trace elements, exhibiting excellent corrosion resistance, heat resistance, and mechanical properties [6]. It possesses a relatively high magnetic permeability, suggesting potential applications in the electromagnetic field, and it is a ferritic stainless steel containing chromium in the range of 12 wt.% to 30 wt.%, where its magnetic permeability varies with the chromium content. Stainless steels with a higher chromium content demonstrate superior corrosion resistance, ductility, and weldability [7]. Additionally, magnetic annealing or the addition of alloying elements (Mo, Si) can influence the microstructure and mechanical properties of 430 stainless steel including its magnetic properties (coercive force); however, the literature on this subject is scarce [8].

Many factors affect electromagnetic characteristics; this paper discusses the effects of annealing and element addition. Relevant studies have explained that electromagnetic materials need more research to integrate electricity, magnetism, and applied mechanics (mechanical properties). In general, 430 stainless steel exhibits magnetic characteristics that differentiate it from other grades of stainless steel, enabling its application in electronic devices, magnetic sensing components, and related fields. However, excessive coercive force in components can lead to delay phenomena during operation [9,10]. Considering the requirement for adequate mechanical strength in electromagnetic steel applications, this study focused on various treatments of 430 stainless steel including magnetic annealing and the addition of different elements (the addition of Mo to form 434 stainless steel or the addition of Si to form KM-31 stainless steel) The incorporation of these two elements is guided by the following studies. Orozco’s research investigated the influence of the addition of molybdenum on the structure and magnetism of magnetic materials. The addition of molybdenum can affect the microstructure, thereby altering magnetic properties. On the other hand, Szymura and Sojka examined the impact of silicon addition on the structure and properties of magnetic materials. The addition of silicon can modify the microstructure and magnetic properties of the alloy [11,12]. In addition to comparing the microstructure and mechanical properties of each system, this study investigated the magnetic properties (hysteresis curve), with the results confirming the influence of metallurgical mechanisms on the magnetic properties of 430 soft magnetic stainless steel.

Electromagnetic steel is used in many fields, and uses electricity to generate magnetism to regenerate force. However, there will always be the problem of the delayed action of the response. At present, the academic community is still unclear regarding this area. This study not only provides a reference for the application of electromagnetic steel, but also integrates materials with electrical applications.

There are relatively few direct literature reports, so the results presented by this study are important. In particular, it has made great contributions to the automation industry.

## 2. Experimental Procedures

This study investigated four types of electromagnetic steel materials (430F, 430F-MA, 434, KM31) (Walsin-Lihwa Corporation, Tainan, Taiwan), with 430F stainless steel chosen as the raw material. The raw material underwent magnetic annealing, with the magnetic annealing conditions set at 800 °C for 4 h in atmospheric conditions, followed by air cooling to room temperature, denoted as 430F-MA. The Curie temperature of 430 stainless steel is approximately 770 °C. In this study, magnetic annealing was conducted at 800 °C. Trace amounts of molybdenum were added to the raw material to form 434 stainless steels. The silicon content of the raw material was increased from 1.0 wt.% to 2.0 wt.%, resulting in the formation of KM31 stainless steel. The chemical compositions of each material are shown in Table 1. Adding Si will reduce the high temperature performance of steel materials, while a high silicon and high chromium content will lead to insufficient ductility. Therefore, this study increased the amount to 1 wt.% Si and reduced the Cr content (forming KM31).

The experiment involved grinding and polishing specimens of 430F, 430F-MA, 434, and KM31, followed by microstructural analysis using a metallographic microscope (OLYMPUS BXX41M-LED, Tokyo, Japan). XRD (Bruker D8D Plus-TXS, Karlsruhe, Germany) analysis of the phase composition was conducted with a 2θ range of 20–90 degrees. Surface elemental distribution analysis of the 430F raw material was performed using EPMA (JEOL JXA-8230, Tokyo, Japan), followed by quantitative composition determination utilizing WDS.

The materials were characterized for mechanical properties using a Vickers hardness tester (Mitutoyo AR-10, Kanagawa, Japan) and tensile testing machine (SHIMADZU AGX-V2, Kyoto, Japan) with a strain rate of 1 × 10^−3^ s^−1^; tensile properties were established through tensile testing. The fracture surface characteristics of the tensile specimens were observed using a scanning electron microscope (SEM, Hitachi 700H, Tokyo, Japan).

Before conducting the magnetic measurements, the specimens were precision-machined into annular samples with inner diameters of 4 mm, outer diameters of 7 mm, and heights of 3 mm. Copper wire coils were wound around the specimens in a ratio of N1:N2 = 15:5, followed by magnetic measurements of the coils. To understand the differences between the direct current (DC) and alternating current (AC) measurements, the 430F specimens were subjected to DC measurements using a soft magnetic alloy direct current B–H curve measurement instrument (Laboratorio Elettrofisico AMH-1K-S, Milan, Italy) and AC measurements using a B–H analyzer (Iwatsu SY-8219, Tokyo, Japan). Subsequently, four types of specimens were analyzed using the B–H analyzer (Iwatsu SY-8219, Japan) under a fixed 1-ampere condition (impressed current) at frequencies of 10 Hz, 100 Hz, and 1000 Hz to establish their magnetic properties. In this study, the small size of the test samples allowed us to distinguish the frequency differences by fixing the applied measuring current at 1-ampere. Each experiment was the average of 3–5 data.

## 3. Results and Discussion

### 3.1. Microstructural Characteristics

**Figure 1:**
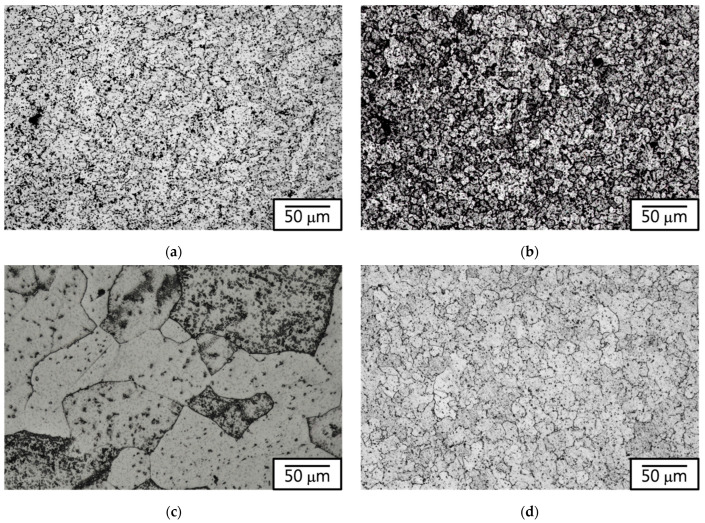
Microstructure of 430 stainless steel: (**a**) 430F (grain size: 11.0 μm), (**b**) 430F after magnetic annealing at 800 °C for 4 h (430F-MA, grain size: 13.0 μm), (**c**) 434 (grain size: 47.0 μm), and (**d**) KM31 (grain size: 26.0 μm).

**Figure 2:**
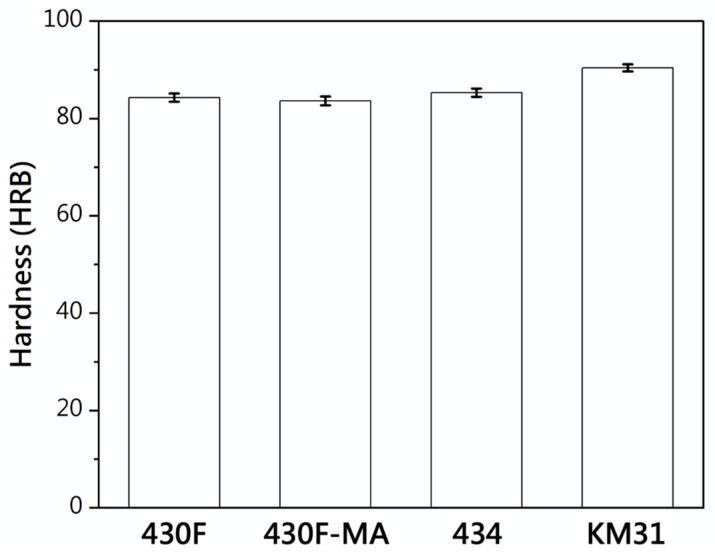
Hardness (HRB) of 430 stainless steel.

The microstructural characteristics of the four electromagnetic steel materials (430F, 430F-MA, 434, and KM31) are depicted in Figure 1. The matrix structures exhibited an equiaxed grain morphology, with the 434 stainless steel matrices displaying the largest average grain size upon measuring the individual base grain sizes (the 434 specimen with Mo did not soften easily, so we increased the annealing temperature to make the grain growth of 430F in the matrix). Hardness measurements of each specimen (Figure 2) revealed that after magnetic annealing, the hardness of 430F-MA was lower than that of the 430F raw material, indicating a softening of the matrix due to the annealing process. Additionally, both the 434 stainless steel and KM31 stainless steel exhibited a higher hardness than the 430F raw material, suggesting that the addition of alloying elements (which suppress the grain boundary movement and the carbide dispersion) strengthens the matrix and reduces the coarse grain effect [13].

**Figure 3:**
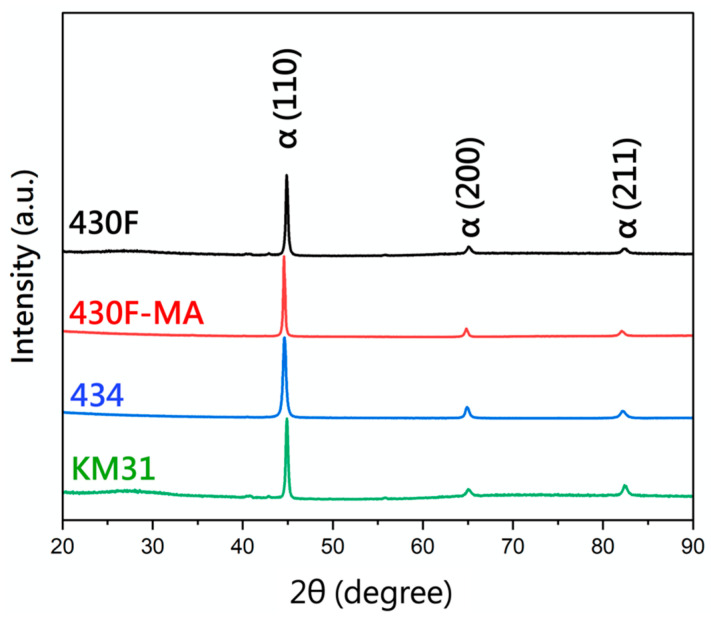
X-ray diffraction pattern of 430 stainless steel.

The phase analysis conducted via X-ray diffraction is presented in Figure 3, confirming that the matrix structures of all four stainless steels were ferritic. Regarding the α (110) peak, all three specimens, 430F-MA, 434, and KM31, exhibited a shift phenomenon, indicating an increase in lattice constant and interplanar spacing [14].

**Figure 4:**
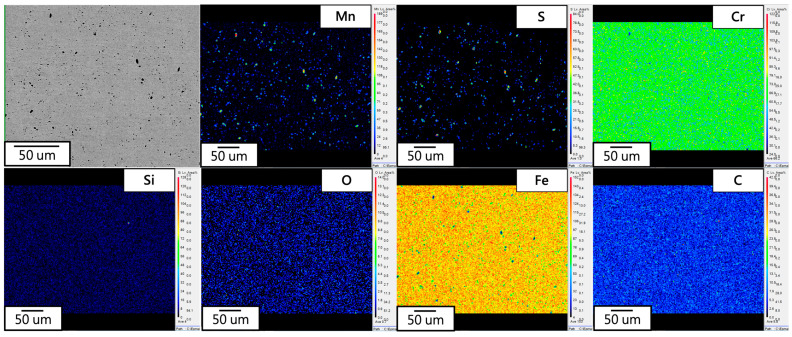
EPMA elemental mapping of 430F.

**Figure 5:**
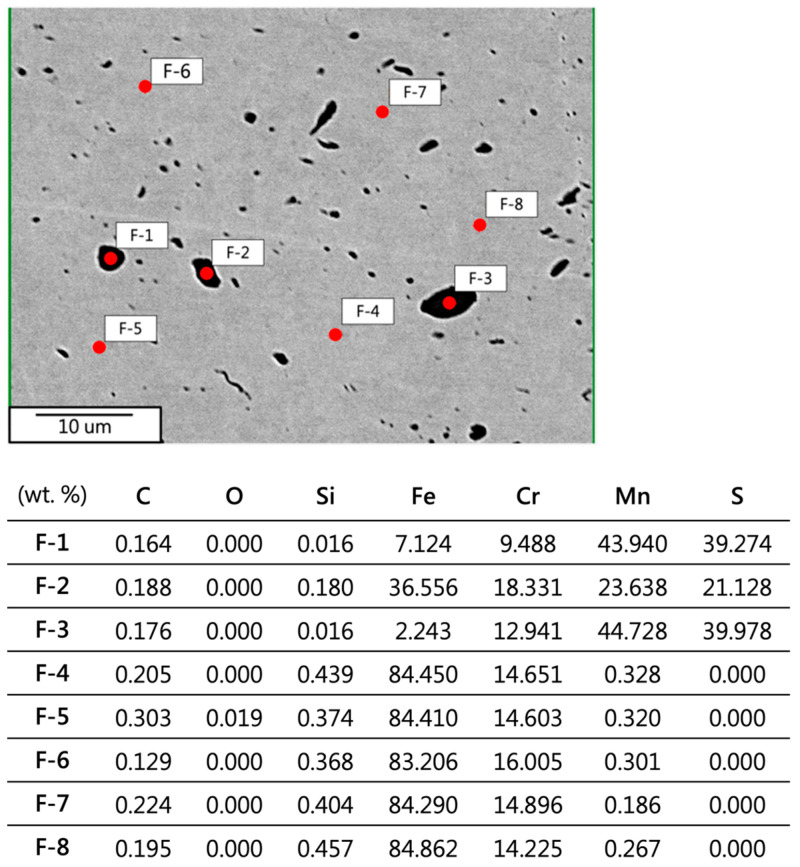
Surface elemental analysis and data of 430F.

The raw material exhibits competitive pricing. In the experiment, the raw material was chosen for elemental analysis. The results of the surface elemental analysis using EPMA are depicted in Figure 4, with analyzed elements including Fe, C, Si, Mn, S, Cr, and O. Image contrast revealed the presence of granular manganese sulfide (MnS) in the matrix. Trace amounts of manganese sulfide can enhance the machinability of electromagnetic steel, while an excess may induce hydrogen embrittlement [15]. The experiment conducted WDS analysis on the second phase in the raw material, with the corresponding positions and phase composition shown in Figure 5, indicating the presence of carbides, MnS, oxides, Fe-Cr compounds, and Cr_3_Si compounds in the matrix. In addition, considering the low C content and bonding energy in an Fe alloy, FeC carbides easily form in electromagnetic steel. SiC (430) and MoC (434) should be the matrix, and the content is lower than that of the FeC carbide.

### 3.2. Tensile Stress–Strain Curves and Tensile Fracture Mechanism

**Figure 6:**
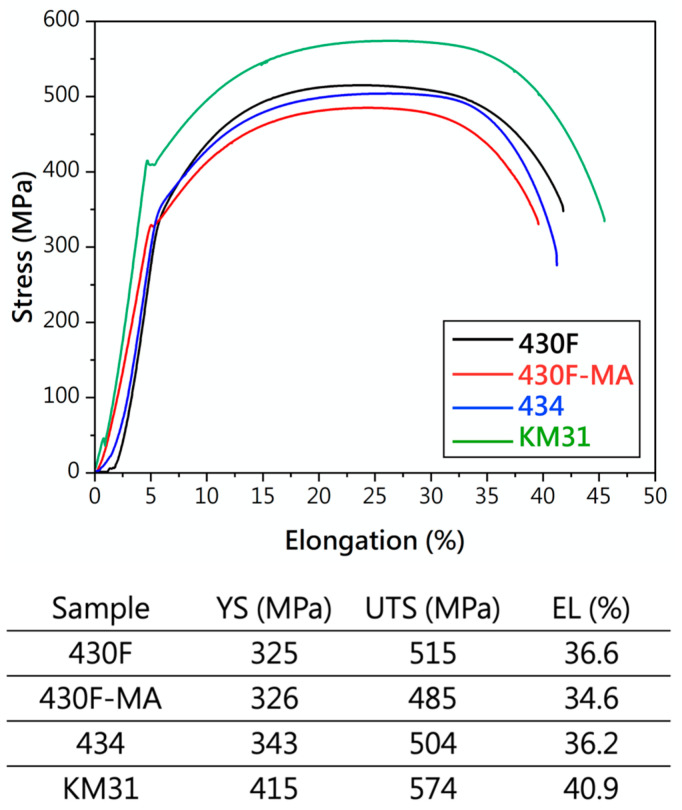
Tensile stress–strain curves and data of 430 stainless steel.

Figure 6 shows the tensile stress–strain curves and data for each specimen, revealing two key characteristics. (1) The raw material (430F) curve exhibited no yield point, while after magnetic annealing, 430F-MA displayed both upper and lower yield points. After magnetic annealing of the raw material, the dislocation density decreased and the slip systems increased, resulting in the appearance of upper and lower yield points in the tensile curve of the sample. Additionally, the curve for KM31 also exhibited upper and lower yield points, indicating a reduction in the deformation slip systems. (2) KM31 demonstrated superior tensile strength and ductility, indicating that the addition of Si can enhance the fracture resistance and improve the fracture toughness. Furthermore, the tensile and elongation properties of the remaining three samples of specimens were comparable.

**Figure 7:**
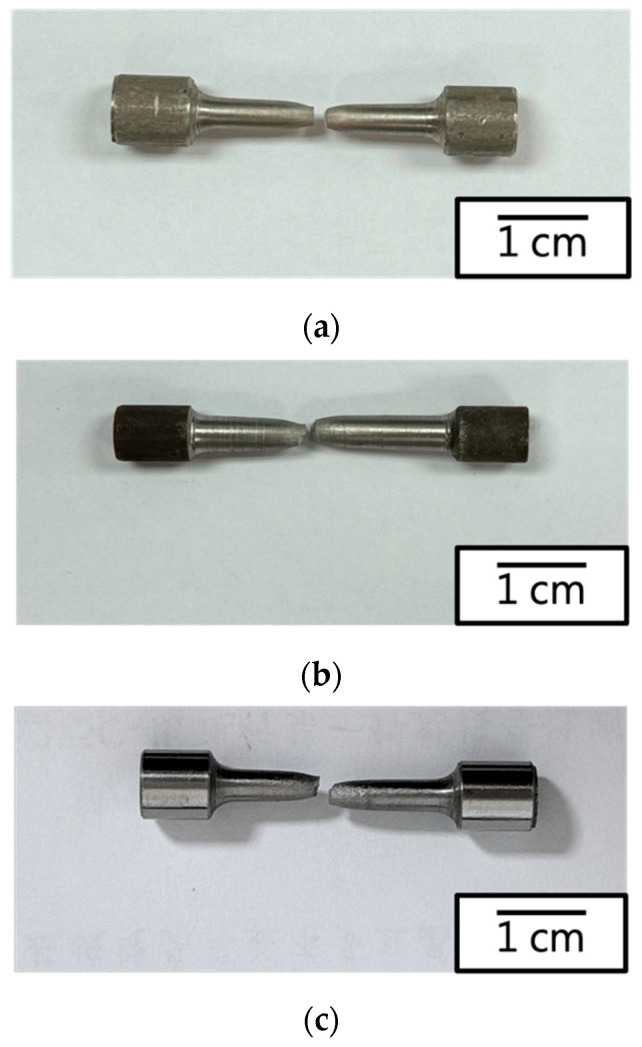

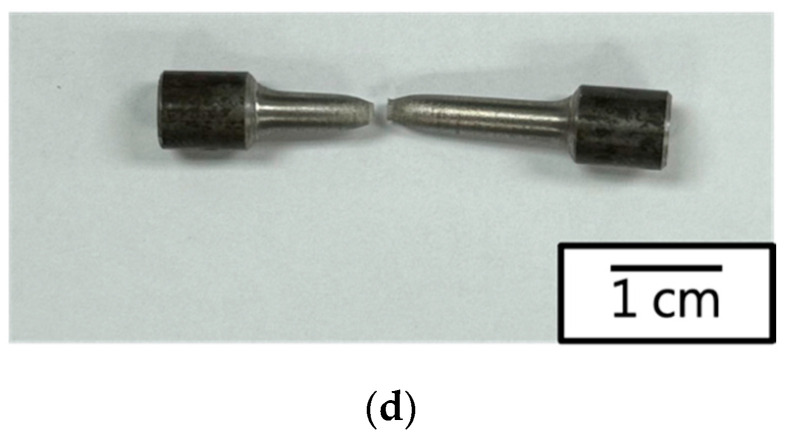
Macroscopic fracture images of the 430 stainless steel tensile sample: (**a**) 430F, (**b**) 430F-MA, (**c**) 434, and (**d**) KM31.

**Figure 8:**
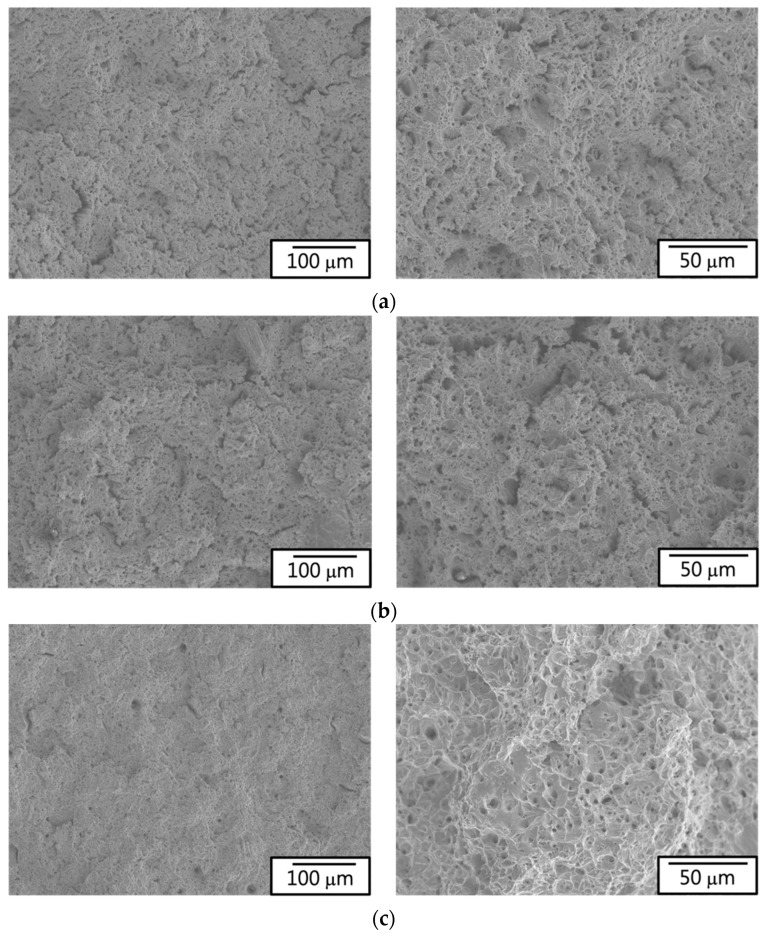

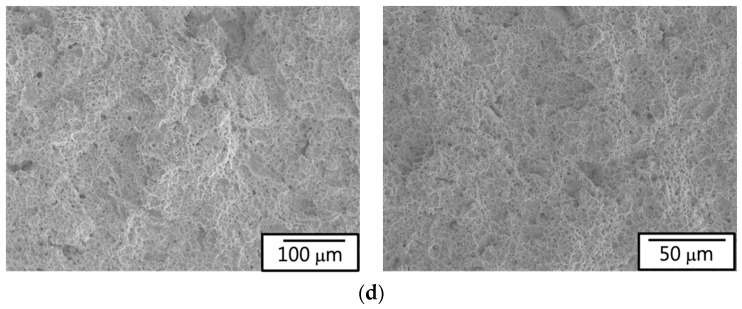
Microstructural of fracture surfaces: (**a**) 430F, (**b**) 430F-MA, (**c**) 434, and (**d**) KM31.

Observation of the macroscopic fracture images for each specimen (Figure 7) revealed ductile fractures, with all fracture surfaces occurring in the parallel region of the test specimens. Figure 8 illustrates the microstructural characteristics of individual fracture surfaces. Figure 8a,b depicts the ductile fracture morphology with numerous small cracks, indicating that the raw material (430F) and the magnetic annealed material (430F-MA) exhibited similar fracture patterns. Furthermore, these fractures showed no significant undulations, with cracks evenly dispersed, confirming that cracks initiate along the grain boundaries in the matrix material during deformation and subsequently propagate to cause failure. It is noteworthy that the examination of the fracture surfaces of specimens with added Mo (434) or high silicon content (KM-31) (Figure 8c,d) revealed numerous dimple features and greater surface undulation. The average size of dimples in Figure 8c was larger than that in Figure 8d, with fewer small cracks, indicating that the specimens with added Mo (434) or high silicon content (KM-31) exhibited preferential deformation fracture within the grains. Based on the tensile curves and data, all four materials demonstrated excellent ductility (elongation > 30% and toughness: integral area under the tensile curve, also known as tensile fracture toughness), making them suitable for application in electromagnetic steel [16]. Considering that the curve for the raw material (430F) exhibited no upper and lower yield point with higher accuracy and reliability, the raw material was chosen for the magnetic property analysis (DC and AC) in the experiment.

### 3.3. Hysteresis Curves and Magnetic Property Analysis

**Figure 9:**
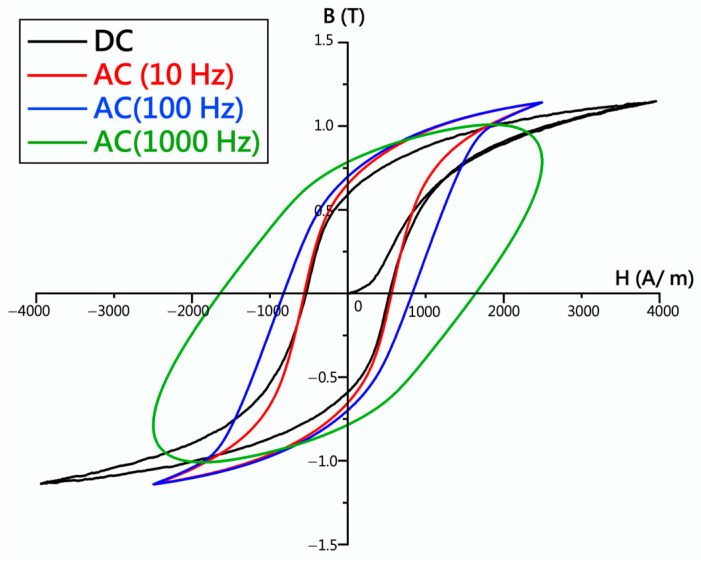
Hysteresis curves of the 430F sample with DC and AC (different frequencies).

**Figure 10:**
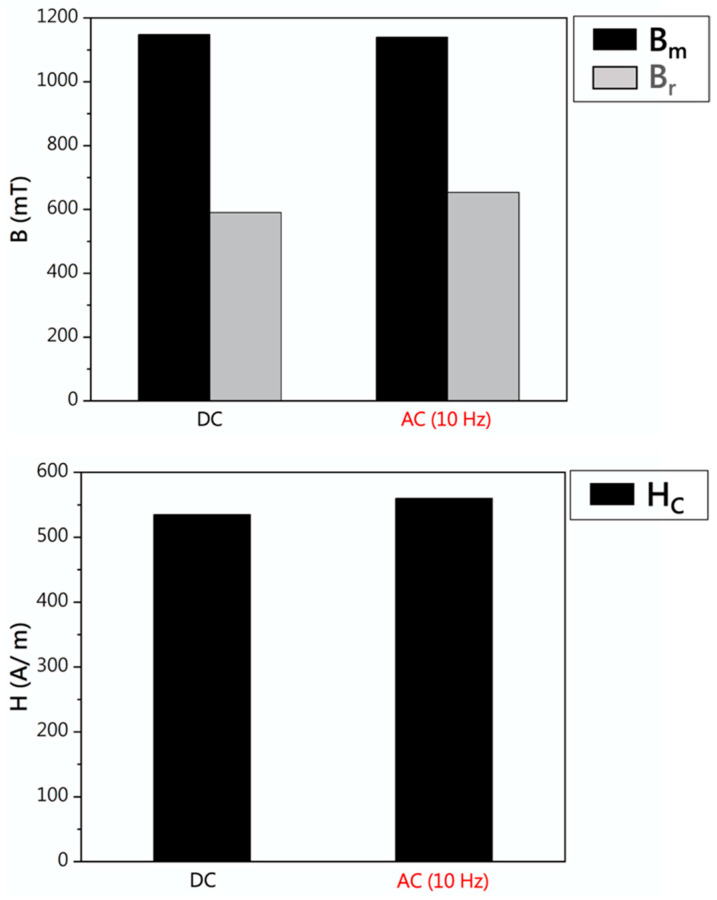
Comparison of the magnetic data of 430F with DC and AC (10 Hz).

Figure 9 depicts the hysteresis curve of the 430F specimen under direct current (DC) and alternating current (AC) (at different frequencies: 10, 100, 1000 Hz). It was observed that the hysteresis curves for DC and AC (10 Hz) magnetization were similar, while the enclosed area of the AC hysteresis loop increased with increasing frequency. Based on the hysteresis curve, the magnetic properties (Bm: maximum magnetization, Br: remanence, Hc: coercive force) under DC and AC (10 Hz) magnetization are shown in Figure 10, indicating the similarity in magnetic properties between the 430F specimen under DC and AC (10 Hz) magnetization [17]. In academia, the hysteresis curves are measured using the AC method, however, in industry, DC is used. Therefore, this result has an application importance.

**Figure 11:**
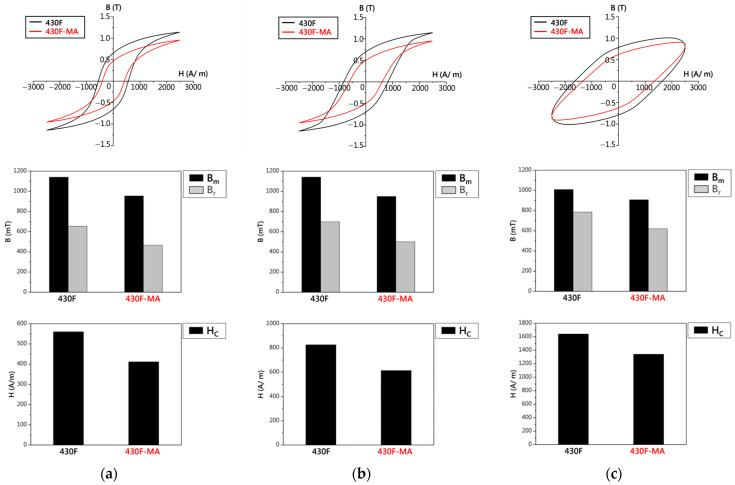
Hysteresis curves and data of 430F and 430F-MA: (**a**) AC-10 Hz, (**b**) AC-100 Hz, and (**c**) AC-1000 Hz.

Figure 11a illustrates the hysteresis curves of the 430F raw material and the magnetic annealed (800 °C—4 h) material 430F-MA under AC-10 Hz conditions. Comparing the magnetic properties of the two materials under AC (10 Hz) conditions, it was found that the magnetic annealing process could reduce the values of Bm, Br, and Hc for 430F [18]. Reducing the remanence and coercive force in soft magnetic stainless steel can help decrease hysteresis losses in applications such as motors or transformers. This indicates that magnetic annealing can alleviate the delay phenomenon in electromagnetic steel operation.

In addition to the AC-10 Hz condition, Figure 11b illustrates the hysteresis curves and magnetic properties of the 430F raw material and the magnetic annealed material 430F-MA under AC-100 Hz conditions. It was observed that the magnetic annealing process could reduce the values of Bm, Br, and Hc for 430F. The effect of magnetic annealing in reducing the values of Bm, Br, and Hc for the 430F raw material persisted even under AC-1000 Hz conditions (Figure 11c). It is noteworthy that with increasing AC frequency, there was a significant increase in Hc values, and under high-frequency conditions, the effect of magnetic annealing on reducing the Hc values was not significant.

**Figure 12:**
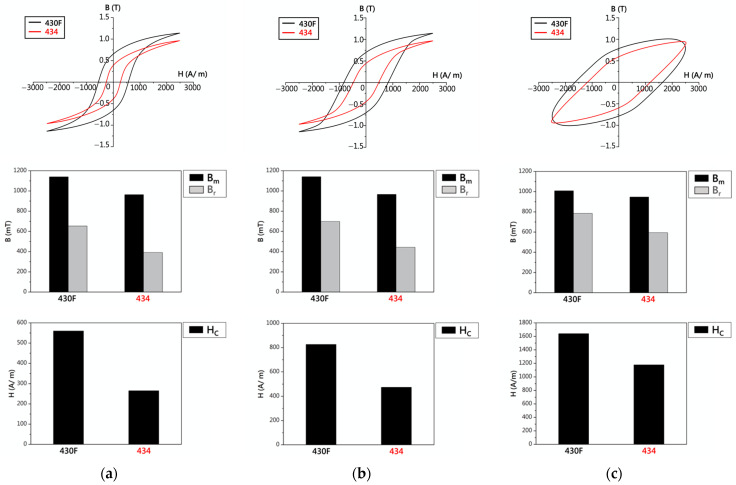
Hysteresis curves and data of 430F and 434: (**a**) AC-10 Hz, (**b**) AC-100 Hz, and (**c**) AC-1000 Hz.

Figure 12 illustrates the hysteresis curves of the 430F raw material and the 434 materials (with added Mo) under AC-10 Hz conditions. Comparing the magnetic properties, it was found that the 434 material exhibited lower values of Bm, Br, and Hc, indicating that the addition of Mo significantly reduced the delay phenomenon in the operation of electromagnetic steel. In addition to the AC-10 Hz condition, Figure 12b displays the hysteresis curves and magnetic properties of the 430F raw material and the 434 materials under AC-100 Hz conditions. The addition of Mo still led to lower values of Bm, Br, and Hc for 430F. Continuing to increase the frequency to 1000 Hz (Figure 12c), the 434 stainless steel exhibited lower values of Bm, Br, and Hc compared to the raw material. Furthermore, when comparing the effects of magnetic annealing (430F-MA) with the addition of Mo (434), it was evident that the addition of Mo significantly reduced the Hc values, confirming that adding Mo elements alters the lattice constant of 430 stainless steel and thus affects its magnetic properties [19].

**Figure 13:**
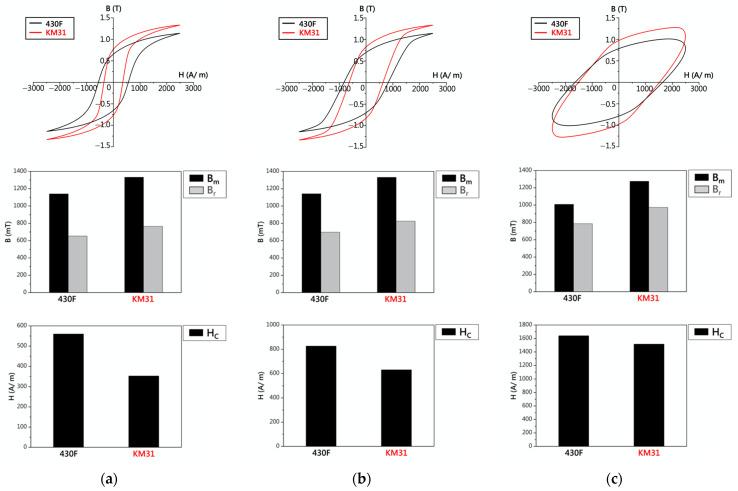
Hysteresis curves and data of 430F and KM31: (**a**) AC-10 Hz, (**b**) AC-100 Hz, and (**c**) AC-1000 Hz.

The hysteresis curves of the 430F raw material and KM31 (with high Si content) under AC-10 Hz conditions are shown in Figure 13a, It was observed that increasing the Si content enhanced the values of Bm and Br for the 430F raw material while reducing the Hc value. Similar results were obtained under AC-100 Hz (Figure 13b) and 1000 Hz (Figure 13c) conditions, indicating that increasing the Si content only lowered the Hc value while increasing the Bm and Br values. An increase in the maximum saturation magnetization for magnetic materials implies that the material can achieve a higher magnetic induction strength under a fixed external magnetic field, thereby enhancing the efficiency of conversion and transmission in magnetic materials. It can be inferred that the presence of a large amount of Si easily forms Cr_3_Si compounds with the Cr, thereby affecting the magnetic properties [20]. The magnetic properties (Bm: maximum magnetization, Br: remanence, Hc: coercive force) of the four sample groups are listed in Table 2, revealing that: (1) Magnetic annealing results in lower Bm, Br, and Hc values, and (2) adding Mo and increasing the Si content have different effects on the magnetic properties.

**Figure 14:**
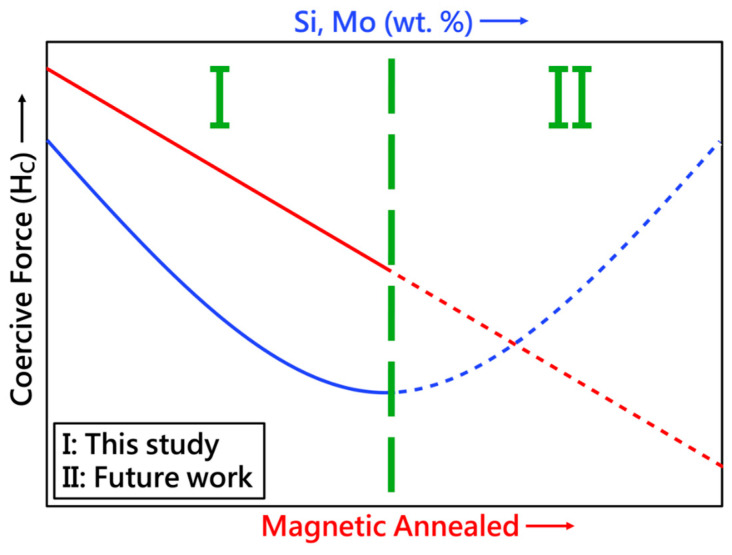
Schematic diagram of the relationship between magnetic annealing and coercive force.

Annealing and alloy addition (Mo or Si) can improve the electromagnetic properties. However, the long duration of annealing and high alloy content will affect the mechanical properties. Figure 14 illustrates the schematic diagram of this study (Zone I). Two characteristics were observed: (1) As the magnetic annealing effect continues, the coercivity force will decrease accordingly, and (2) as the number of added elements (Si, Mo) increases, the coercivity force will decrease accordingly. However, there will be a turning point when adding a certain number of elements, leading to the appearance of an inflection point. The study of this phenomenon (Zone II) will be conducted in future works.

## 4. Conclusions

The four electromagnetic steel materials (430F, 430F-MA, 434, and KM31) exhibited equiaxed grain matrix structures. Magnetic annealing, the addition of Mo, and increased Si content all led to an increase in lattice constant and interplanar spacing. The second phases present in the raw material included carbides, MnS, oxides, Fe-Cr, and Cr_3_Si.

The tensile properties and elongation of the samples were similar, with KM31 demonstrating superior tensile strength and ductility. The addition of Si enhanced the fracture resistance and improved its fracture toughness. Both the raw material (430F) and magnetic annealed material (430F-MA) exhibited similar ductile fracture patterns. The addition of Mo (434) or high silicon content (KM31) led to deformation and fracture predominantly at the grain level during tensile testing (by suppressing grain boundary movement and carbide dispersion), resulting in fewer microcracks and the appearance of numerous dimple features on the fracture surface.

The magnetic properties of the 430F sample under DC and AC-10 Hz conditions were similar. At different AC frequencies (10, 100, 1000 Hz), magnetic annealing and the addition of Mo could reduce the values of Bm, Br, and Hc for the 430F raw material. Increasing the Si content could decrease the Hc value while increasing the Bm and Br values. The presence of a large amount of Si tends to form Cr_3_Si compounds with the matrix Cr, thereby affecting the magnetic properties.

Relevant studies have suggested that electromagnetic materials need more research to integrate electricity, magnetism, and applied mechanics (mechanical properties). It is confirmed that magnetic annealing with appropriate alloy additions is positive for electromagnetic properties.

## 5. Limitations

To investigate the magnetic annealing effect of 430 stainless steel, subsequent studies will explore the influence of different annealing temperatures and times on the magnetic properties. Additionally, in future research, transmission electron microscopy (TEM) will be utilized to analyze the metallurgical effects of Si, aiming to elucidate the magnetic properties of low-Si (430F), medium-Si (430FR), and high-Si (KM31) electromagnetic stainless steels.

## Figures and Tables

**Table 1:** Chemical composition of stainless steel (wt.%).

	C	Si	Mn	P	S	Cr	Mo
430F	0.12	1.00	1.25	0.06	0.15 min	16–18	-
430F-MA	0.12	1.00	1.25	0.06	0.15 min	16–18	-
434	0.12	1.00	1.25	0.04	0.03	16–18	1.0
KM31	0.12	2.00	1.25	0.06	0.15 min	13	-

**Table 2:** Magnetic data of stainless steel.

**10 Hz**	**430F**	**430F-MA**	**434**	**KM31**
Bm (mT)	1140	954	963	1332
B_r_ (mT)	653	466	391	765
H_c_ (A/m)	560	411	265	353
**100 Hz**	**430F**	**430F-MA**	**434**	**KM31**
B_m_ (mT)	1141	949	966	1331
B_r_ (mT)	698	500	443	825
H_c_ (A/m)	826	613	474	630
**1000 Hz**	**430F**	**430F-MA**	**434**	**KM31**
B_m_ (mT)	1009	907	947	1275
B_r_ (mT)	785	620	594	973
H_c_ (A/m)	1640	1339	1177	1516

## Data Availability

The original contributions presented in the study are included in the article, further inquiries can be directed to the corresponding author.

## References

[1] Lula R.A. (1985). Toughness of Ferritic Stainless Steels.

[2] Beddoes J., Parr J.G. (1999). Introduction to Stainless Steels.

[3] Baddoo N.R. (2008). Stainless steel in construction: A review of research, applications, challenges and opportunities. J. Constr. Steel Res..

[4] Cao M.S., Wang X.X., Zhang M., Shu J.C., Cao W.Q., Yang H.J., Yuan J. (2019). Electromagnetic response and energy conversion for functions and devices in low-dimensional materials. Adv. Funct. Mater..

[5] Fiorillo F., Bertotti G., Appino C., Pasquale M. (2016). Soft magnetic materials. Wiley Encyclopedia of Electrical and Electronics Engineering.

[6] Cheng X., Jiang Z., Wei D., Zhao J., Monaghan B.J., Longbottom R.J., Jiang L. (2015). High temperature oxidation behavior of ferritic stainless steel SUS 430 in humid air. Met. Mater. Int..

[7] Yu Y., Shironita S., Souma K., Umeda M. (2018). Effect of chromium content on the corrosion resistance of ferritic stainless steels in sulfuric acid solution. Heliyon.

[8] Ku W., Ge F., Zhu J. (1997). Effect of magnetic field annealing on the giant magnetoimpedance in FeCuMoSiB ribbons. J. Appl. Phys..

[9] Oxley P., Goodell J., Molt R. (2009). Magnetic properties of stainless steels at room and cryogenic temperatures. J. Magn. Magn. Mater..

[10] Ara K. (1989). Magnetic characteristics of ferromagnetic stainless steels. IEEE Trans. Magn..

[11] Orozco C., Melendez A., Manadhar S., Singamaneni S.R., Reddy K.M., Gandha K., Ramana C.V. (2017). Effect of molybdenum incorporation on the structure and magnetic properties of cobalt ferrite. J. Phys. Chem. C.

[12] Szymura S., Sojka L. (1986). The effect of silicon on the structure and properties of Fe-Cr-Co permanent magnet alloys. J. Magn. Magn. Mater..

[13] Zhang G.J., Lin X.H., Liu G., Zhang N.N., Sun J. (2011). The influence of silicon content on microstructure and hardness of Mo-Si alloys. Int. J. Refract. Met. Hard Mater..

[14] Jing S.-Y., Lin L.-B., Huang N.-K., Zhang J., Lu Y. (2000). Investigation on lattice constants of Mg-Al spinels. J. Mater. Sci..

[15] Pressouyre G.M., Blondeau R., Cadiou L. (1984). HSLA steels with improved hydrogen sulfide cracking resistance. J. Mater. Energy Syst..

[16] Poulnikov A., Permiakov V., De Wulf M., Dupré L., Makaveev D., Melkebeek J. (2002). Investigation of residual effects of tensile stress on magnetic properties of non-oriented electrical steel. IEEE Trans. Magn..

[17] Ton M., Fortenbery B., Tschudi W. (2008). DC Power for Improved Data Center Efficiency.

[18] Phan M.H., Peng H.X., Wisnom M.R., Yu S.C., Chau N. (2006). Effect of annealing on the microstructure and magnetic properties of Fe-based nanocomposite materials. Compos. Part A Appl. Sci. Manuf..

[19] Dudognon J., Vayer M., Pineau A., Erre R. (2008). Mo and Ag ion implantation in austenitic, ferritic and duplex stainless steels: A comparative study. Surf. Coat. Technol..

[20] Zhang X.M., Dai X.F., Jia H.Y., Chen G.F., Liu H.Y., Luo H.Z., Wu G.H. (2012). Electronic structures and magnetism of Cr3Z (Z = Si, Ge, Sb) with DO3 structures. Comput. Mater. Sci..

